# EXPANDING SOCIAL TIES AS A GOAL IN LATER LIFE: THE ROLE OF SOCIAL NETWORK CHARACTERISTICS

**DOI:** 10.1093/geroni/igz038.623

**Published:** 2019-11-08

**Authors:** Danielle Oleskiewicz, Karen Rook

**Affiliations:** University of California, Irvine, Irvine, California, United States

## Abstract

Older adults often winnow their social ties to focus on emotionally rewarding ties (Charles & Carstensen, 2010). Some older adults, however, have small social networks that preclude much winnowing or aversive social ties from which disengagement is difficult. These individuals might be motivated to expand, rather than contract, their social ties. The current study sought to extend knowledge regarding potential links between social network characteristics and older adults’ interest, effort, and success in creating new social ties. We expected that small social networks and negative social ties might motivate interest and effort directed toward forming new social ties but that positive social ties might foster success in efforts to form new ties. In-person interviews were conducted with participants (N = 351, Mean age = 74.16) in a larger study of older adults’ social networks and well-being. The interviews assessed participants’ social networks, as well as their interest, effort, and success in making new social ties. Participants’ social network composition, rather than size, was associated with greater motivation to establish new social ties. Negative social ties were associated with greater interest and effort directed toward forming new social ties. Positive social ties were related to greater success (due, in part, to their support provision) and, unexpectedly, were also related to greater interest and effort directed toward forming new ties. Older adults sometimes seek to expand, rather than contract, their social ties, and characteristics of their social networks appear to play a role in fueling and influencing the success of such efforts.

